# Topical antibiotic therapy in eye infections - myths and certainties in the era of bacterial resistance to antibiotics

**Published:** 2020

**Authors:** Victoria Aramă

**Affiliations:** * ”Carol Davila” University of Medicine and Pharmacy, Bucharest, Romania; Prof. Dr. Matei Balș National Institute of Infectious Diseases, Bucharest, Romania

**Keywords:** eye infections, topical antibiotic therapy, antibiotic resistance

## Abstract

Globally, the alarming increase in the rate of antibiotic (AB) resistance of bacteria is currently considered one of the 7 major threats to the human race along with terrorism, nuclear proliferation and pollution. Judicious use of AB by physicians in all medical and surgical specialties is essential to limit the extent of resistance to AB.

In Europe, Romania ranks among the first in terms of the rate of resistance to AB of the main bacteria involved in eye infections (EI).

The principles of a judicious antibiotic therapy in ophthalmology are: performing the bacteriological determinations necessary to establish the bacterium involved in EI and its sensitivity to AB; avoiding the treatment of viral infections with AB; knowledge of the local rate of resistance of bacteria to AB; first choice of an AB with a spectrum appropriate to the aetiology of EI; the chosen AB must penetrate well into the eye tissues; using the local route of administration whenever possible; avoiding sub-dosing and shortening the duration of antibiotic therapy; abandoning the “myth” that a *“in vitro”* bactericidal AB would be inherently more clinically effective (*“in vivo”*) than a bacteriostatic AB; requesting the consultation of infectious diseases for EI with AB multidrug-resistant bacteria. The available ophthalmic topics contain antibiotics from the following classes: aminoglycosides, fluoroquinolones, chloramphenicol, glycopeptides, polymyxins, etc. The increase in the fluoroquinolone resistance rate of the bacteria involved in EI has recently led to the recommendation that, in the absence of the antibiogram, it is best to avoid first-line antibiotic therapy with topical fluoroquinolones alone in keratitis.

**Abbreviations**:

AB = antibiotic, AG = aminoglycosides, AUC = area under the curve, Cf = chloramphenicol, Cmax = maximum concentration in tears, CNS = central nervous system, CSF = cerebrospinal fluid, DNA = deoxyribonucleic acid, ECDC = European Centre for Disease Prevention and Control, EI = eye infections, ENT = ear, nose and throat, EU = European Union, FQ = fluoroquinolones, HSV = Herpes simplex virus, MBC = minimum bactericidal concentration, MIC = minimum inhibitory concentration, MRSA = methicillin-resistant S. aureus, MRSE = methicillin-resistant S. epidermidis, MSSA = methicillin-sensitive S. aureus, MSSE = methicillin-sensitive S. epidermidis, PCR = polymerase chain reaction, S = sulfonamides, SPC = summary of product characteristics, USA = United States of America, VZV = Varicella zoster virus

## Introduction

Globally, the alarming increase in the rate of bacterial resistance to AB is currently considered one of the 7 major threats to the human race, along with terrorism, nuclear proliferation and air pollution [**1**]. Judicious use of AB by physicians in all medical and surgical specialties is essential to limit the extent of resistance to AB.

In Europe, Romania unfortunately ranks among the first in terms of the rate of resistance to AB of the main bacteria involved in eye infections [**1**]. That is why we considered it useful to offer a set of clarifications for ophthalmologists, in a way which addresses the principles of judicious antibiotic therapy in eye infections (EI), in the context of the alarming increase in bacterial resistance to AB.

## *Clinical classification of eye infections accessible to topical antibiotic therapy*.

Eye infections accessible to a topical treatment with AB are superficial infections of the anterior pole: conjunctivitis, keratitis and blepharitis [**2**,**3**].

Risk factors that favor the appearance of severe forms of conjunctivitis and keratitis are: age extremes, unbalanced diabetes, immunosuppression, underlying ophthalmic pathology (sicca syndrome, corneal dystrophy, corneal graft, recent eye surgery, contact lens wear, tear duct obstruction, disorders of eyelid static, synophthalmia) and local corticosteroid therapy [**2**,**3**].

## Acute conjunctivitis 

Acute conjunctivitis is the inflammation of the conjunctiva in the absence of corneal damage. Most conjunctivitis is bilateral. They may have a viral, allergic aetiology or are associated with sicca syndrome [**2**,**3**].

Acute conjunctivitis is clinically manifested by painless diffuse conjunctival hyperaemia (“pink eye”), abundant tearing, conjunctival discomfort (feeling of “sand in the eye”), with conjunctival secretions that agglutinate the eyelashes, without affecting visual acuity and with benign evolution [**2**,**3**].

Sampling of conjunctival secretions for bacteriological examination is not routinely performed in mild to moderate acute conjunctivitis, but only in patients with severe forms or risk factors for severe forms (diabetes, immunosuppression), as well as those with therapeutic failure in primary therapy intent. Gram-stained smears, cultures or molecular biology (PCR) determinations can be performed from conjunctival secretions to identify nucleic acids specific to different aetiological agents [**2**,**3**].

## Acute keratitis

*Viral acute keratitis* it is usually superficial and consists in the appearance of dendritic corneal ulcerations, highlighted by fluorescein staining [**2**,**3**]. 

*Bacterial acute keratitis* is a diffuse or localized corneal infection, which, if not treated immediately, can have a serious impact on visual acuity. It is clinically manifested by diffuse and painful conjunctival hyperaemia, usually unilateral, accompanied by photophobia and excessive tearing [**2**,**3**].

There are 3 distinct clinical forms of acute keratitis: simple or punctate keratitis, corneal ulcer and corneal abscess [**2**,**3**].

*Simple acute keratitis and corneal ulcers* are either the early stages of a presuppurative superficial bacterial infection, or correspond to a toxic, traumatic or inflammatory aggression [**2**,**3**]. 

*Corneal abscesses* correspond to a deep and suppurative bacterial infection. They usually occur after neglected traumatic corneal ulcers; the latter often being caused by wearing contact lenses. The severity criteria of corneal abscesses are: location in the optic axis, diameter over 3 mm, stromal infiltration, inflammation of the anterior chamber, clinical worsening after 24 hours of appropriate topical antibiotic treatment [**2**,**3**].

Bacteriological examination is not necessary in superficial corneal abscesses, but should always be performed in deep corneal abscesses or those located in the optic axis. The bacteriological diagnosis in acute keratitis involves the collection of a corneal sample, by corneal scraping, by the ophthalmologist [**2**,**3**]. From the corneal secretions, gram-stained smears, cultures or molecular biology determinations (PCR) can be performed to identify nucleic acids specific to different aetiological agents [**2**,**3**]. 

## Aetiology of eye infections accessible to topical antibiotic therapy

The normal conjunctival commensal microbiota is composed of gram-positive bacteria in a proportion of over 70% (coagulase-negative staphylococcus, *Staphylococcus aureus*, group A, B, C, G and D streptococci, pneumococcus and non-groupable streptococci) [**2**,**3**].

In contact lens wearers, the commensal flora is clearly dominated by gram-negative bacteria (*Proteus spp., Haemophilus spp., Pseudomonas spp., Klebsiella spp.*, etc.) [**2**,**3**]. 

A monocentric retrospective study performed in a hospital in Turin evaluated, between 1988 and 2017, the dynamics of the bacterial aetiology of eye infections (EI) and the AB resistance profile for isolated bacteria [**4**]. More than 15,500 bacterial strains, isolated and identified from patients with conjunctivitis, keratitis and endophthalmitis, were included in the study. Gram-positive bacteria accounted for 73.5% of the isolated strains, the most commonly identified being coagulase-negative staphylococcus, *Staphylococcus aureus*, pneumococcus and various species of streptococci. Gram-negative bacteria accounted for about 25% of the isolated strains, the most commonly identified being *Pseudomonas aeruginosa, E. coli, K. pneumoniae, Proteus spp and H. Influenzae* [**4**]. Fluoroquinolones (FQ) and chloramphenicol (Cf) have been shown to be the most effective in vitro AB against bacteria involved in EI, followed by tetracyclines, ampicillin and aminoglycosides (AG) [**4**]. The highest rate of multidrug resistance was detected in enteric gram-negative bacilli and coagulase-negative staphylococci [**4**]. In dynamics, there was an increase in the resistance rate of gram-negative bacteria to AG and gram-positive bacteria (especially *Staphylococcus aureus*) to FQ and AG [**4**]. Locoregional surveillance of the aetiology and susceptibility to AB of bacteria involved in EI is crucial in establishing empirical (first-line) antibiotic treatment in EI [**4**]. 

## Aetiology of acute conjunctivitis

Viruses dominate the aetiology of acute conjunctivitis, the most often involved being adenoviruses and enteroviruses. Outbreaks of haemorrhagic acute conjunctivitis are described, which are usually caused by adenovirus 11, enterovirus 70, or Coxsackie A24 virus [**2**,**3**]. 

Bacterial aetiology should be suspected in case of purulent conjunctival secretions. The bacteria most commonly involved in adults are *Staphylococcus aureus* and pneumococcus, and in children *H. influenzae* [**2**,**3**]. 

Acute conjunctivitis and blepharitis in people without contact lenses are usually caused by gram-positive bacteria, especially staph. In contact lens wearers, acute conjunctivitis is usually produced by gram-negative bacteria, including *Pseudomonas aeruginosa*. In the case of infants with lacrimal duct imperfections, acute conjunctivitis is usually produced by bacteria from the commensal flora of the ENT (ear, nose and throat) area (pneumococcus, streptococci, *Haemophilus influenzae*) [**2**,**3**]. 

## Aetiology of acute keratitis

The aetiology of acute keratitis can be viral (HSV1, HSV2, VZV, adenoviruses) or bacterial. The involvement of fungi is more common in immunocompromised patients and is suggested by the existence of traumatic corneal lesions caused by plant-origin foreign bodies [**2**,**3**].

The bacteria most commonly involved in keratitis in patients without contact lenses are staphylococcus (60% of cases) and streptococcus (16% of cases), while gram-negative bacilli predominate in contact lens wearers (*Klebsiella spp, Serratia spp, Pseudomonas aeruginosa*). In keratitis encountered in children, bacteria from the commensal flora of the ENT area (pneumococcus, *Haemophilus influenzae*) predominate [**2**,**3**]. 

*Resistance to antibiotics of bacteria involved in eye infections*.

## Bacterial resistance to antibiotics in Europe

**Fig. 1:**
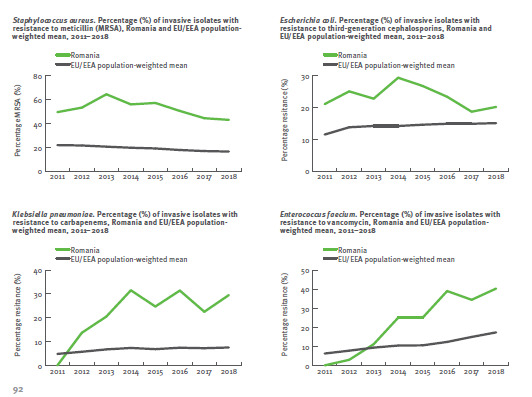
Dynamics of the antibiotic resistance rate in Romania, compared to the European average, between 2011 and 2018, according to ECDC [**1**]

**Fig. 2:**
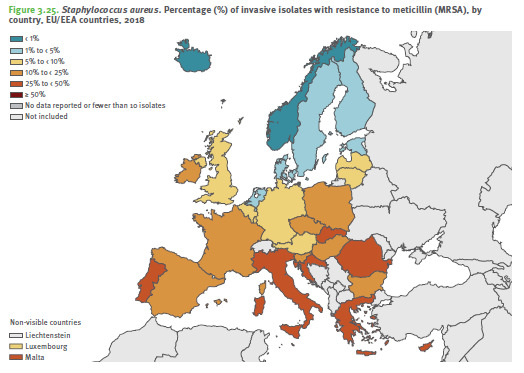
ECDC - methicillin resistance rate of invasive strains of *Staphylococcus aureus* (MRSA) in Europe, 2018 [**1**]

**Fig. 3:**
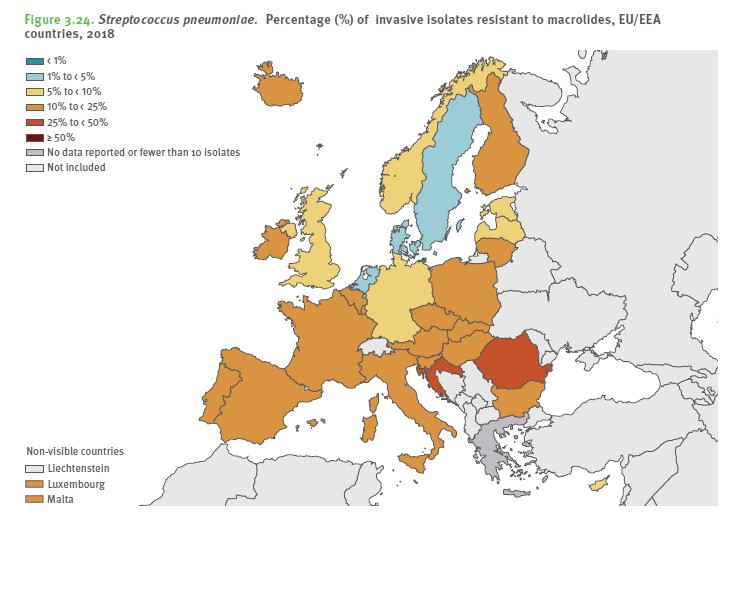
ECDC - macrolide resistance rate of invasive pneumococcal strains in Europe, 2018 [**1**]

According to the 2018 Annual Report of the ECDC on bacterial resistance to antibiotics (AB) in the EU, Romania unfortunately has resistance rates well above the European average for methicillin-resistant *S. aureus* (MRSA), *E. coli* resistance to 3rd generation cephalosporins, *K. pneumoniae* resistance to carbapenems and *E. fecalis* resistance to vancomycin (**Fig. 1**) [**1**]. **Fig. 2** shows the rate of methicillin resistance of invasive *Staphylococcus aureus* strains (MRSA) compared to EU countries, and **Fig. 3** illustrates the macrolide resistance rate of invasive pneumococcal strains [**1**]. One can observe that Romania has significantly higher resistance rates than the rest of the EU countries.

## Bacterial resistance to antibiotics in Romania

**Fig. 4:**
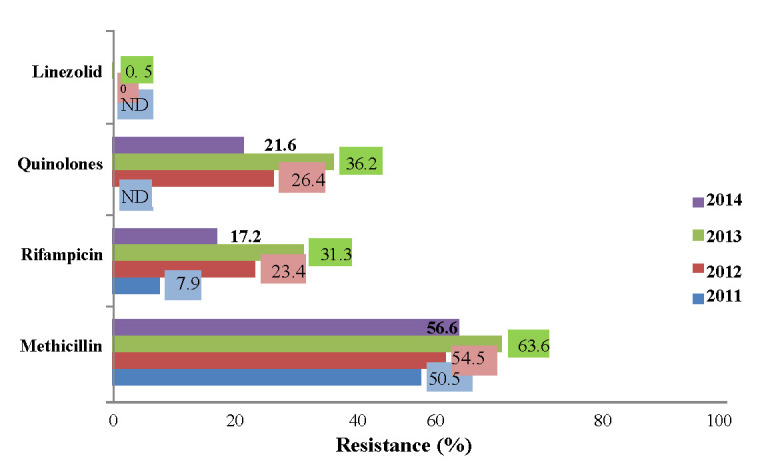
The evolution of *S. aureus* resistance to antibiotics between 2011 and 2014 [**5**]

The National Center for Surveillance and Control of Communicable Diseases monitors the dynamic evolution of AB resistance of the main bacteria involved in human infections. **Fig. 4** shows the evolution of antibiotic resistance of *Staphylococcus aureus* between 2011 and 2014 [**5**]. Over 55% of *S. aureus* strains were resistant to methicillin (MRSA) and over 20% were resistant to FQ [**5**].

Romania - resistance of *S. aureus*

Report by Popescu G. *et al.*, 2016 [**5**]

• 417 strains tested for Meticillin: 236 resistant (56.5%)

• 396 strains tested for Rifampicin: 68 resistant (17.2%)

• 417 strains tested for Fluoroquinolones: 90 resistant (21.6%)

## Resistance of bacteria involved in eye infections to topical ophthalmic antibiotics

Despite high concentrations in ocular tissues obtained after topical administration of AB, more and more clinical failures have been reported in recent years following the empirical use of topical FQ in ocular infections, some of which are due to bacterial resistance to FQ.

A study published in 2017, which assessed the dynamics of the aetiological profile and resistance to AB for bacteria involved in EI in the USA between 2005 and 2015, showed an increasing trend (by 8.8%) of the involvement of gram-positive bacteria and decrease (by 2.8%) of the involvement of gram negative bacteria (**Table 1**) [**6**]. 

Depending on the location of EI, the most frequently isolated bacteria are presented in **Table 1** [**6**]. 

**Table 1:** The most frequently isolated pathogens from EI in the USA between 2011 and 2015 [**6**]

Top 10	Eye infections overall (n = 4649)	Conjunctivitis (n = 876)	keratitis (N = 1498)	Endophthalmitis (n = 198)
1	*S. aureus* 1027 (22.1%)	*S. aureus* 311 (25.5%)	*P. aeruginosa* 405 (27%)	*S. epidermidis* 60 (30.3%)
2	*P. aeruginosa* 639 (13.7%)	*H. influenzae* 65 (7.4%)	*S. aureus* 234 (15.6%)	*S. viridans* 28 (14.1%)
3	*S. epidermidis* 312 (6.7%)	*P. aerugiosa* 55 (6.3%)	*Fusarium spp* 117 (7.8%)	*Candida spp* 18 (9.1%)
4	*S. viridans* 222 (4.8%)	*Adenovirus* 43 (4.9%)	*Serratia spp* 78 (5.2%)	*S. aureus* 15 (7.6%)
5	*S. marcescens* 177 (3.8%)	*S. viridans* 39 (4.5%)	*S. viridans* 63 (4.2%)	Coagulase-Negative Staphylococci 11 (5.6%)
6	*Fusarium spp* 175 (3.8%)	*C. trachomatis* 33 (3.8%)	*S. epidermidis* 59 (3.9%)	
7	*S. pneumoniae* 113 (2.4%	*S. pneumoniae* 32 (3.7%)	HSV1 56 (3.7%)	
8	*H. influenzae* 113 (2.4%)	*Candida spp* 22 (2.5%)	*S. pneumoniae* 39 (2.6%)	
9	*C. albicans* 92 (2%)	*Corynebacterium* 20 (2.3%)	*C. albicans* 31 (2.1%)	
10	*Corynebacterium spp* 67 (1.4%)	*Serratia spp* 20 (2.3%)	*Acanthamoeba spp* 30 (2%)	

It could be observed that the bacteria most frequently involved in EI were: *S. aureus, P. aeruginosa, S. epidermidis*, viridans streptocci and pneumococci.

The rate of methicillin resistance of *S. aureus* (MRSA) was 42.1% and that of *S. epidermidis* (MRSE) was over 46.30%. MRSA and MRSE strains had higher rates of resistance to other ABs compared to methicillin-sensitive strains of *S. aureus* (MSSA) and *S. epidermidis* (MSSE). Thus, only 19% of MRSA strains were sensitive to ciprofloxacin, compared to 80% of MSSA strains. Ciprofloxacin-resistant MRSA strains also had cross-resistance to levofloxacin and moxifloxacin [**6**]. 

It was observed that over 90% of the gram-negative bacteria isolated were sensitive to amikacin, gentamicin and tobramycin [**6**].

This study showed a continuous increase in the rate of resistance to FQ of the following bacteria isolated from EI: MSSA, MRSE, MRSA, pneumococcus, viridans streptococci, *H. influenzae* and *P. aeruginosa*. Practically, in this study the empirical antibiotic therapy with FQ covered less than 75% of the strains of the 3 most frequently isolated bacteria from EI (*S. aureus*, coagulase-negative staphylococci and *P. aeruginosa*), which suggested that in a quarter of cases, the empirical initiation of topical FQ therapy might result in clinical failure [**6**].

Numerous studies have reported that MRSA strains have a much higher rate of resistance to other classes of antibiotics than MSSA, the most affected being FQ [**6**-**8**]. Table 2 shows how the susceptibility to ciprofloxacin decreases from 87% for MSSA to 27.9% for MRSA, this decrease being more important in the case of keratitis (from 79.8% to 5.2%). Unfortunately, the same dramatic decrease in susceptibility is observed for levofloxacin and moxifloxacin, which leads to the conclusion that topical FQ alone should no longer be recommended for empirical (first-line) antibiotic therapy for severe EI, especially concerning keratitis [**6**]. In countries where the use of topical FQ in EI is much more widespread (India, Brazil), the FQ resistance rate of MSSA and MRSA rises to over 70%, and of *P. aeruginosa* and *E. coli* to 30% [**6**].

**Table 2** also shows that the decrease in sensitivity to aminoglycosides of MRSA compared to MSSA is much more discrete, from 98.5% to 90.9% [**6**]. The susceptibility rate for vancomycin is similar to MSSA and MRSA. In countries where the topical versions of aminoglycosides (tobramycin, gentamicin, netilmicin) have been used extensively as first line therapy in EI, an increase in the resistance rate of staphylococci (especially coagulase-negative staphylococci), which can reach 30%, has been observed [**6**].

In the same study, the susceptibility rate of *P. aeruginosa* was 97% for ciprofloxacin and levofloxacin, 95% for imipenem, 99.5% for tobramycin and 96.5% for ceftazidime [**6**].

**Table 2:** Susceptibility rate to other AB of MRSA vs. MSSA isolated from ocular infections [**6**]

	Eye infections overall n (% susceptibility)	conjunctivitis n (% susceptibility)	keratitis n (% susceptibility)
Ciprofloxacin			
MSSA	604 (87.4%)	164 (89.4%)	144 (79.8%)
MRSA	416 (27.9%)	108 (20.7%)	93 (5.2%)
Total	1020	272	237
Gentamicin / Tobramycin			
MSSA	604 (98.5%)	164 (97.6%)	144 (98.6%)
MRSA	416 (90.9%)	109 (87.9%)	93 (88.3%)
Moxifloxacin			
MSSA	312 (89%)	106 (89.4%)	86 (81%)
MRSA	375 (23.8%)	103 (20.4%)	86 (5.7%)
Total	687		
Levofloxacin			
MSSA	604 (89.1%)	164 (91.8%)	144 (84%)
MRSA	416 (27.9%)	108 (22.5%)	93 (5.2%)
Total	1020		
Vancomycin			
MSSA	604 (99%)	164 (99%)	144 (98%)
MRSA	416 (97%)	109 (96%)	93 (98%)
Total	1020		
% MRSA	40.8%	39.9%	39.2%

Therefore, the experts’ recommendations are that in areas with a high incidence rate of MRSA, first-line empirical therapy should use combinations of 2 topics with antibiotics (FQ and vancomycin) or a topic that is active on MRSA. However, the use of antibiotic combinations would have the disadvantage of the selection pressure of resistant bacterial strains. Another recommendation of experts is to use various classes of AB as first-line therapy, in turns, precisely to limit the extension of the rate of resistance to a certain class of AB, as it happened with FQ.

In this context, topical chloramphenicol, which unlike FQ and AG was much less used in EI, but which has a very wide spectrum, a low resistance rate and good ocular penetrability, should be reconsidered and recommended in first-line empirical therapy of EI.

## Topical antibiotics used in the treatment of eye infections

Topical ophthalmic treatments include eye drops, ointments and AB gels. They are prescribed by ophthalmologists, but as well by family doctors, paediatricians and doctors from other specialties. Topical ophthalmic ABs are prescribed for both curative and preventive purposes, for EI prophylaxis after ophthalmic surgery.

In superficial EI, topical antibiotic therapy provides good bioavailability at the ocular surface, equal to or higher than systemic antibiotic therapy and allows the treatment of most infections of the anterior ocular pole. Topical ophthalmic antibiotic therapy may promote the selection of bacterial strains with mutations of resistance to AB, especially when treatment is prolonged. Therefore, topical antibiotic therapy should be short-term [**3**].

## Pharmacokinetic features of topical ophthalmic antibiotics

In the case of antibiotic eye drops, in the form of instilled drops in the conjunctival sac, the preparation is diluted in the tear film, distributed over the entire ocular surface and in contact with the superficial layer of the cornea (corneal epithelium) and the conjunctiva, which coat the eyeball and the inner face of the eyelids.

The AB concentration in tears decreases progressively over time, the causes being the following: dilution in the tear film, resorption at the level of the conjunctiva, elimination through the tear duct and penetration through the cornea into the anterior chamber [**3**].

An AB penetrates the cornea better, the more it is in a viscous support (which increases the remanence time), the more lipophilic it is and the lower its molecular weight, (which allows it to cross the cell barrier more easily) [**3**]. The presence of continuity solutions at the corneal level will increase the penetration rate of AB [**3**].

The parameters that characterize topical AB kinetics at the ocular level are: the area under the curve of the AB concentration in tears (AUC); maximum concentration in tears (Cmax); corneal penetration to the anterior chamber; the systemic passage of AB [**3**].

The goal of a topical antibiotic therapy is to obtain effective concentrations (higher than the MIC and lower than the toxic concentration), while the AB remains a maximum time duration in contact with the eye. This maximum contact duration depends on the viscosity of the topic, the class of AB contained in the eye, the pH, the osmotic concentration and the type of adjuvants contained in the preparation [**3**]. These parameters are especially important for time-dependent AB and to a lesser extent for concentration-dependent AB (AG, FQ), for which the Cmax/ MIC and AUC/ MIC ratio is decisive [**3**]. Based on these pharmacokinetic data, AB gels and ointments are considered to have a longer remanence and a more potent and prolonged antibacterial effect [**3**].

## Topical ophthalmic fluoroquinolones (FQ) 

FQs are synthetic ABs with small molecules that interfere with bacterial DNA synthesis, inhibiting a bacterial enzyme (DNA gyrase) that ensures that bacterial DNA is supercoiled inside the bacterial cell [**2**]. In the absence of this enzyme, the bacterial DNA will no longer be coiled, reaching the entire cell and breaking the cell membrane. FQs have an *in vitro* bactericidal effect and a broad antibacterial spectrum, being active on both gram-positive and gram-negative bacteria. Unfortunately, they have limited activity on multidrug-resistant staphylococci, on streptococci, on enterococci, but also on *Acinetobacter* spp, *Stenotrophomonas matophila, Burkholderia cepacian*, etc. [**3**,**4**,**6**-**8**]. 

FQ are effective in severe pathologies, such as corneal abscesses. Due to the increased risk of selection of resistant strains, the current ECDC recommendations on the judicious use of topical AB state that FQ should be avoided in mild forms of anterior ocular pole infections, for which there are other therapeutic alternatives. They are indicated in severe forms of conjunctivitis, keratitis, corneal ulcers, and ciprofloxacin is indicated in corneal abscesses [**3**,**4**,**6**-**8**]. The concentrations of ciprofloxacin in the cornea are effective against most of the bacteria involved, because the CMI90 is less than 1 microgram/ ml, except for the following bacteria: MRSA, *Bacteroides fragilis*, pneumococcus, some streptococci, enterococci, *Acinetobacter* spp [**3**,**4**,**6**-**8**]. After systemic administration (oral or injectable), FQ can have serious side effects: musculoskeletal, cardiovascular (QT prolongation), hepatic cytolysis, digestive dysbiosis (including pseudomembranous colitis with *C. difficile*), allergic, photosensitization [**2**]. Following topical administration, systemic absorption is reduced and the risk of systemic adverse reactions to topical FQ is negligible. Allergic reactions and photosensitization may occur. FQ are contraindicated in pregnant women and children under 14 years old.

## Topical ophthalmic aminoglycosides (AG)

AG are ABs with bactericidal action in vitro, which inhibits the synthesis of bacterial proteins in the ribosomal fractions 30 and 50S. They have a relatively narrow spectrum, being active on gram-negative bacilli (including *P. aeruginosa*), gram-positive cocci. They are inactive on anaerobic bacteria [**3**]. AG molecules have low penetrability and diffusibility when applied topically, and after oral administration they are not absorbed from the digestive tract [**2**]. The penetration of AG through the cornea to the anterior chamber is modest, and the concentrations achieved in the anterior chamber are infratherapeutic, which can lead to the rapid appearance of resistant bacterial strains [**3**]. Administered by injection, AGs have poor tissue penetration into the lungs and do not penetrate the CSF, CNS, bone and eyes, which is why it is recommended that, in systemic infections, AG should be used only in combinations of AB. Injectable AG may have severe side effects:

- Reversible nephrotoxicity, which is why the doses of AG should be adjusted to creatinine clearance, the duration of treatment should not exceed 7 days, and the patient’s kidney function should be closely monitored during therapy with AG [**2**]. 

- Ototoxicity is irreversible and cumulative over time, which is why treatments with AG should be short-lived and not repetitive [**2**].

- The curarization effect (neuromuscular block) may occur after rapid intravenous administration. Therefore, systemic AG are contraindicated in patients with myasthenia gravis and in those under curarization [**2**].

- Allergic reactions, up to anaphylactic shock. 

Administered topically, the systemic absorption of AG is very low, which is why the risk of nephrotoxicity after topical administration is purely theoretical [**3**]. AG are contraindicated in pregnant women because they can cause cochleovestibular toxicity in the foetus.

## Ophthalmic topic chloramphenicol (Cf) 

Cf has *in vitro* bacteriostatic or bactericidal action depending on the bacterium and the concentration achieved at the site of infection [**2**]. Thus, it has *in vitro* bactericidal effect on *S. pneumoniae* and bacteriostatic effect on *S. aureus* and streptococci. Cf acts by inhibiting the synthesis of bacterial proteins in 50S ribosomal fractions. It has a broad spectrum, being active on both gram-positive and gram-negative bacteria, both aerobic and anaerobic bacteria and is also active on atypical bacteria (*Chlamydia* spp, *Rickettsia* spp, *Mycoplasma* spp) [**2**]. Administered orally, Cf has a very good digestive absorption, an excellent tissue diffusion (including in the CNS, CSF, lymph nodes, eyes) and penetrates well intracellularly [**2**]. Acquired resistance to Cf is rarely reported. Cf has a very good intraocular penetration. Systemic Cf use has declined sharply in recent decades due to fears of a haematological toxic effect (aplastic anaemia) (1/ 60,000 oral or injectable administrations) [**2**]. It is considered that the penetration into the systemic circulation of Cf administered as an ophthalmological topic is insignificant and that it cannot lead to the appearance of this haematological toxic effect [**3**,**9**,**10**]. To date, there are no reports of cases of aplastic anaemia in which a cause-and-effect relationship with the administration of topical preparations of Cf could be certainly demonstrated [**9**,**10**]. The existence of a genetic determinism has been demonstrated, which favors the appearance of this hematotoxic effect (after systemic administration) only in some patients [**9**,**10**]. Given the advantages of this antibiotic (low cost, very affordable, broad spectrum, good activity on bacterial strains resistant to other topical AB, good intraocular penetration), Cf continues to be indicated as first-line treatment in numerous international therapeutic guidelines for superficial anterior pole eye infections [**3**,**9**,**10**]. As a precaution, it is recommended to use topical Cf preparations for short periods of time [**3**,**9**,**10**]. For added safety, topical Cf should be avoided in patients with haematological diseases. Cf is contraindicated in pregnant women, infants and young children due to the possibility of “gray baby syndrome” in the newborn [**2**].

## Sulfonamides (S)

Sulfonamides inhibit tetrahydrofolic acid synthesis, eventually blocking bacterial DNA replication [**2**]. They have a broad spectrum, but unfortunately, due to their excessive use in the past, the rate of bacterial resistance to S is high in Romania and their use involves identifying the bacterium involved and its sensitivity to AB. In addition, S have a high potential for hypersensitivity, including after topical administration, with a risk of severe allergic reactions (Stevens Johnson syndrome). They have good penetrability in tissues, including the CNS, prostate, lung and biological fluids [**2**]. 

**Topical preparations containing combinations of 2 AB** have the advantage of extending the antibacterial spectrum and of a synergistic effect, but they have the disadvantage of increasing the risk of selecting AB-resistant strains [**3**].

Topical ophthalmic preparations with AB available in Romania are presented in **Table 3**.

**Table 3:** Topical preparations with antibiotics available in Romania (according to the SPCs of the products)

Trade name, Presentation form	Active substance	Detailed composition	Indications	Contraindications	Dosage	Side effects	The manufacturing company, Year of placing on the market
**Betabioptal**, Ophthalmic drops	betamethasone, chloramphenicol	1 ml of eye drops contains: 2 mg betamethasone + 5 mg chloramphenicol + thiomersal	Non-purulent ophthalmic infections, with chloramphenicol-sensitive bacteria, when betamethasone has anti-inflammatory action. Inflammation of the anterior pole, especially postoperatively, bacterial and allergic conjunctivitis, acute iridocyclitis	Known hypersensitivity to one of the components Ocular hypertension Conjunctivitis and herpes keratitis (HSV, VZV) Fungal or mycobacterial infections History of haematological diseases	1-2 drops in the conjunctival sac, 3-6 times/ day	Local irritation Local allergic reactions Increased eye pressure Sub-capsular cataract (after prolonged treatment)	THEA FARMA, 2006
**TOBRADEX**, Ophthalmic drops	dexamethasone, tobramycin	1 ml of eye drops contains: 3 mg tobramycin + 1 mg dexamethasone + Benzalkonium chloride	Eye inflammation that responds to corticosteroid treatment, associated with superficial bacterial infections: eyelid and bulbar conjunctivitis, keratitis, anterior uveitis, corneal lesions due to chemical burns, heat, radiation or foreign body penetration Prevention and treatment of inflammation after cataract surgery in adults and children over 2 years of age.	Known hypersensitivity to one of the components Herpes conjunctivitis and keratitis (HSV, VZV) Fungal or mycobacterial infections Purulent infections	1-2 drops in the conjunctival sac, 4-6 times/ day After cataract surgery 1 drop x 4 times/ day, starting preoperatively with 1 day and continuing postoperatively for up to 23 days	Eye pain Increased eye pressure Local irritations Allergic reactions Thinning of the cornea at risk of perforation after prolonged treatment	Alcon, 2005
**Dexatobrom**, Ophthalmic drops	dexamethasone, tobramycin	1 ml of eye drops contains: 3 mg tobramycin + 1 mg dexamethasone + Benzalkonium chloride	Anti-inflammatory and prophylactic treatment in infections with tobramycin-sensitive bacteria, after cataract surgery and in situations where corticosteroid treatment is required and there is a risk of superficial eye infections	Known hypersensitivity to one of the components Herpes conjunctivitis and keratitis (HSV, VZV) Fungal or mycobacterial infections Purulent eye infections Children	1 drop in the conjunctival sac every 4-6 hours	Blurred vision after applying the drops Intraocular hypertension Posterior subcapsular cataract Local irritations Allergic reactions Secondary fungal infections	Rompharm, 2009
**Floxal**, Ophthalmic drops	ofloxacin	1 ml of eye drops contains: 3 mg ofloxacin + Benzalkonium chloride	Infections of the anterior segment caused by ofloxacin-sensitive bacteria: conjunctivitis, keratitis, blepharitis, external eyelid stye, chalazion, corneal ulcer	Known hypersensitivity to one of the components	1 bit in the conjunctival sac 4 times a day, maximum 14 days.	Local irritation Local hypersensitivity reactions Systemic hypersensitivity reactions (rare)	Gerhard Mann Chem-Pharm Factory GmbH-Bausch & Lomb, 2004
**L-optic**,Ophthalmic drops	levofloxacin	1 ml of eye drops contains: 5 mg levofloxacin hemihydrate + Benzalkonium chloride	Treatment of external bacterial infections in adults and children over 1 year of age caused by levofloxacin-sensitive bacteria	Known hypersensitivity to levofloxacin or other quinolones	Initially 1-2 drops in the conjunctival sac at 2 hours (but not more than 8 administrations/ day) and then 4 times/ day for 5 days.	Local irritation Local allergic reactions Systemic allergic reactions (rare)	Rompharm, 2014
**Netildex**,Ophthalmic drops	dexamethasone, netilmicin	1 ml of eye drops contains: 1.32 mg dexamethasone + 4.55 mg netilmicin + Benzalkonium chloride	Inflammatory diseases of the anterior ocular pole, including postoperative, in the presence or when there is a risk of a bacterial eye infection	Known hypersensitivity to one of the components Ocular hypertension Glaucoma Herpes eye infections Fungal or mycobacterial infections Lactation	In adults: 1 drop in the conjunctival sac 4 times/ day	Local irritation Increased intraocular pressure Posterior subcapsular cataract	Sifi, 2006
**Quixin**,Ophthalmic drops	Levofloxacin 0.5%	1 ml of eye drops contains: Levofloxacin 0.5% (5 mg/ ml) + Benzalkonium chloride	Treatment of conjunctivitis caused by sensitive bacteria	Known hypersensitivity to levofloxacin or other quinolones	Initially, in the first 2 days of treatment: 2 drops in the conjunctival sac every 2 hours (maximum 8 times/ day) Then, in the next 5 days, 2 drops every 4 hours (maximum 4 times/ day)	Transient decrease in vision Local irritation Local allergic reactions	Santen, In the process of registration in Romania
**Kanamycin sulphate 1%**, Ointment	Kanamycin sulphate	100 g of ophthalmic ointment contain: 1 g of kanamycin monosulphate + Lanolin + Vaseline	Treatment of external infections of the eyeball and appendages produced by kanamycin-sensitive bacteria: conjunctivitis, keratitis, corneal ulcer, blepharitis, blepharoconjunctivitis, dacryocystitis.	Known hypersensitivity to kanamycin or to any of the excipients	Apply in the conjunctival sac 3-4 times/ day.	Mild and transient eye irritation.	SC Antibiotics, 2004
**Vigamox**, Ophthalmic drops	moxifloxacin	1 ml contains: 5.45 mg moxifloxacin hydrochloride	Purulent bacterial conjunctivitis caused by sensitive bacteria	Known hypersensitivity to moxifloxacin or other fluoroquinolones	In adults: 1 drop 3 times/ day for 7-8 days	Local allergic reactions Systemic allergic reactions (rare) Local irritations	Alcon/ Novartis, 2010
**Maxitrol**, Ophthalmic drops	dexamethasone, Neomycin sulphate, Polymyxin B sulphate	1 ml contains: 1 mg dexamethasone + 3500 UI neomycin sulphate + 6000 UI polymyxin B sulphate + Benzalkonium chloride	Inflammatory eye diseases associated with superficial infections with sensitive bacteria Pathological conditions at risk of bacterial infections	Hypersensitivity to one of the components Herpes eye infections Fungal or mycobacterial eye infections Untreated purulent infections	In adolescents and adults: 1-2 drops 4-6 times/ day	Local allergic reactions Local irritation Posterior subcapsular cataract Glaucoma	Novartis, 2005

Unfortunately, the available topical ophthalmic preparations in Romania contain antibiotics only from a few classes: aminoglycosides, fluoroquinolones, chloramphenicol, neomycin and polymyxin B. In other countries, there are topical preparations with antibiotics from other classes: fusidic acid, tetracycline, vancomycin, bacitracin and so on.

**Topical preparations containing combinations of AB and steroidal or non-steroidal anti-inflammatory drugs** are recommended for bacterial infections with an important inflammatory component. If it is only an inflammatory pathology, a topic with anti-inflammatory is used and the use of the topic antibiotic should be avoided [**3**].

**The “myth” of clinical superiority of a bactericidal AB vs. a bacteriostatic AB**

The “myth” of the clinical superiority of a bactericidal (BC) AB vs. a bacteriostatic (BS) AB dates back in classical bacteriology, but today, modern bacteriology recommends abandoning this myth because the intrinsic clinical superiority of BC antibiotics has not been demonstrated compared to BS [**11**-**13**]. 

“Bactericidal” and “bacteriostatic” are two notions defined *“in vitro”* (in laboratory conditions) by specific parameters: 

- MIC (Minimum Inhibitory Concentration) defines bacteriostasis and represents the lowest concentration of AB, which inhibits visible bacterial growth on culture medium [**2**].

- MBC (Minimum Bactericidal Concentration) defines bactericide and is the lowest concentration of AB that destroys 99.9% of bacteria [**2**].

The BC and BS effect are abstract notions, which have no clinical relevance, because in the laboratory, the pharmacokinetic and pharmacodynamic properties of AB, the degree of penetration and concentration of AB at the site of infection and the intracellular penetration of antibiotics cannot be taken into account [**11**-**13**]. Therefore, the “bacteria-antibiotic couple” behaves differently in the test tube filled with culture media than in the body, which means that the antibacterial efficacy of an AB measured *“in vitro”* (in the laboratory) is not equivalent to clinical efficacy. In fact, in clinical practice, cases of therapeutic failure are frequently reported when using an AB that proved to be active *in vitro*, as well as cases of therapeutic success with an AB that proved inactive *in vitro*, which is called the “paradox between *in vitro* and *in vivo”*.

Thus, the myth of the superiority of BC antibiotics is only speculative, as there is no scientific evidence to support a superior intrinsic clinical efficacy of BC antibiotics compared to BS in immunocompetent patients with infections located in tissues and organs. The use of antibiotics with BC effect is recommended in severe systemic infections (sepsis, meningitis, endocarditis) and in immunocompromised patients [**11**-**13**]. 

Although aminoglycosides are BC in vitro, they do not concentrate well in tissues, achieving low concentrations in the lung, CNS, bone or eye, which is why their *in vivo* clinical efficacy is often lower than a BS antibiotic that concentrates well at the site of infection (e.g. macrolides) [**11**-**13**]. 

Although they are BS *in vitro*, macrolides and chloramphenicol concentrate very well in tissues, which is why their *in vivo* clinical efficacy is often superior to BC antibiotics [**11**-**13**]. 

Depending on the concentration achieved at the site of infection, some AB can be both BC and BS:

- Macrolides are BS but achieve alveolar fluid concentrations up to 40 times higher than the serum ones, which are bactericidal for most bacteria involved in pneumonia. For this reason, in international guidelines, macrolides are considered AB of first intention in pneumonia.

- Chloramphenicol is BS, but it achieves bactericidal concentrations in tissues.

In infections caused by bacteria with intracellular development (*Chlamydia* spp, *Ricketssia* spp, *Mycobacteria*) treatment should include AB that have the ability to penetrate intracellularly (macrolides, cyclins, rifampicin, fluoroquinolones, etc.). AB that cannot penetrate intracellularly (beta-lactams) should be avoided [**11**-**13**]. 

At this time, it is considered that other properties of an AB (penetration and concentration at the site of infection, binding to serum proteins) are much more important for therapeutic success than its BC or BS effect [**13**].

## Recommendations for therapeutic guidelines for the topical treatment of eye infections. Treatment of acute bacterial conjunctivitis 

The evolution of bacterial acute conjunctivitis is usually favorable under standard treatment, which consists of conjunctival lavages with saline and the use of topical antiseptics. In severe forms or in the presence of risk factors for severe forms (unbalanced diabetes, immunosuppression, recent ophthalmic surgery), antibiotic topics are required [**2**,**3**]. The severity criteria are: rich purulent secretions, chemosis, corneal oedema, eyelid oedema, intense pain, significant tearing, decreased visual acuity, photophobia [**2**,**3**]. 

In the era of bacterial resistance to AB, international guidelines recommend that in mild and moderate acute conjunctivitis, only conjunctival lavage with saline and topical antiseptics without topical AB should be performed in patients without risk factors for severe forms.

Although clinical studies have shown that the addition of a topical antibiotic is accompanied by a faster improvement of symptoms in mild and moderate acute conjunctivitis, it is recommended to avoid topical AB in acute conjunctivitis without signs of severity, occurring in patients without risk factors for severe forms [**3**]. The extra comfort brought by the topical AB in an acute conjunctivitis without signs of severity is strongly counterbalanced by the risk of selecting resistant bacterial strains, which, from an individual risk, turns into a collective risk, because it can also affect people around [**3**]. 

In underdeveloped countries, where the hygienic-sanitary conditions are precarious, it is preferred to use a topical AB in acute conjunctivitis, in order to avoid severe corneal complications, generating blindness [**3**].

The antibacterial efficacy of various topical ABs for ophthalmic use is broadly similar, but international guidelines recommend that topical FQ alone is avoided in first-line therapy, for fear of the increased risk of selecting resistant strains. Topical FQs are reserved for severe forms of acute conjunctivitis and situations in which there has been a therapeutic failure in another topical AB initially administered [**3**,**6**-**8**]. 

If the dosage and method of administration are followed, the probability of selecting resistant bacterial mutants after topical treatment with AB is theoretically low, because the use of ophthalmic topic agents achieves high local concentrations of AB. The use of an ophthalmic topic with sub-dosed AB and long-term and repetitive topical treatments can cause the appearance of resistant bacterial strains in the commensal flora, which will replace the sensitive ones. In recent years, there has been an increase in the incidence of eye infections with multi-resistant bacteria (MRSA, *Pseudomonas aeruginosa*), which are difficult to treat [**3**,**6**-**8**]. 

## Treatment of acute bacterial keratitis 

Antibiotic therapy is mandatory in bacterial acute keratitis, which must benefit from early and adequate antibiotic treatment, because untreated corneal abscess can progress on short term to corneal perforation and endophthalmitis, and on long term to definitive corneal opacity [**3**].

Topical antibiotic therapy at usual doses penetrates with difficulty into corneal abscesses, requiring eye drops with a higher concentration of AB than usual, which can be prepared in hospital pharmacies [**3**]. 

In the absence of severity criteria or risk factors for severe forms, bacterial acute keratitis can be treated on an outpatient basis, with topical AB preparations, alone or in combination of two AB [**3**]. 

For severe forms of bacterial acute keratitis and for those who failed in the initial empirical antibiotic therapy, it is recommended to hospitalize and repeat the bacteriological examination, with the adaptation of antibiotic therapy according to the antibiogram, often requiring combinations of 2-3 topical AB, with antibiotic concentrations higher than the usual ones. In severe forms, systemic antibiotic therapy should be combined with topical antibiotic therapy [**3**].

For corneal abscesses, ulcers and keratitis caused by gram-negative bacilli and methicillin-sensitive staphylococcus (MSSA or MSSE), topical fluoroquinolone preparations (ciprofloxacin, levofloxacin) are recommended as a first option [**3**]. If the involvement of a methicillin-resistant staphylococcus (MRSA, MRSE) is suspected or confirmed, it is recommended to use combinations of ophthalmic topics that include vancomycin, as a significant percentage of MRSA and MRSE is resistant to FQ. Unfortunately, there are no vancomycin topics available in Romania, which is why the choice of antibiotic must be adapted according to the result of the antibiogram.

For uncomplicated keratitis, corneal ulcers and abscesses produced by proven sensitive bacteria, topical preparations with aminoglycosides (tobramycin, netilmicin, kanamycin), polymyxin B, rifampicin are recommended [**3**].

In acute keratitis, topical corticosteroid therapy is contraindicated in the absence of adequate etiological therapy. The use of local anaesthetics is not recommended in keratitis.

## Conclusions

Instead of conclusions, we propose the characteristics of a topical antibiotic ideal for eye infections: broad spectrum of activity (gram-positive and gram-negative bacteria), including antibiotic-resistant bacteria (MRSA, MRSE); bactericidal or bacteriostatic; can achieve increased concentration in the eye tissues (cornea, aqueous humor); has reduced systemic absorption and negligible risk of toxic effects; has low probability of inducing resistance mutations; is well tolerated locally; can also be administered to children.

In order to optimize the management of eye infections, it is very important to know their aetiology and to know the local sensitivity profile of bacteria to antibiotics, in order to guide the choice of initial antibiotic therapy. We must first consider the local rate of staphylococcal resistance to methicillin, because MRSA has high rates of resistance to other classes of antibiotics, such as fluoroquinolones. The dosage for each antibiotic must be observed, in order to avoid sub-dosing, which favors the appearance of resistance. In order to avoid the selection pressure for resistant bacterial mutants, it is important not to use the same class of antibiotics constantly in all patients.
